# A Multiview SAR Target Recognition Method Using Inner Correlation Analysis

**DOI:** 10.1155/2021/9703709

**Published:** 2021-11-24

**Authors:** Lei Lei, Dongen Guo, Zhihui Feng

**Affiliations:** ^1^School of Computer and Software, Nanyang Institute of Technology, Nanyang 473000, China; ^2^College of Information and Management Science, Henan Agricultural University, Zhengzhou 450002, China

## Abstract

This paper proposes a synthetic aperture radar (SAR) image target recognition method using multiple views and inner correlation analysis. Due to the azimuth sensitivity of SAR images, the inner correlation between multiview images participating in recognition is not stable enough. To this end, the proposed method first clusters multiview SAR images based on image correlation and nonlinear correlation information entropy (NCIE) in order to obtain multiple view sets with strong internal correlations. For each view set, the multitask sparse representation is used to reconstruct the SAR images in it to obtain high-precision reconstructions. Finally, the linear weighting method is used to fuse the reconstruction errors from different view sets and the target category is determined according to the fusion error. In the experiment, the tests are conducted based on the MSTAR dataset, and the results validate the effectiveness of the proposed method.

## 1. Introduction

With the continuous enhancement of synthetic aperture radar (SAR) data acquisition capabilities, it has become possible to acquire SAR images of the same target from multiple views, which provides more available information for correct target recognition [1]. Early SAR target recognition methods were mainly based on single-view SAR images, that is, to determine the target category in a single SAR image through effective feature extraction [2–18] and classification [19–32] with the support of training samples. There are relatively few research studies on target recognition methods based on multiview SAR images, whose basic idea can be divided into two categories [33–43]. One type considers that the SAR images from various views are relatively independent. So, the multiple views can be merged through the algorithm of parallel decision-making. In [33], researchers made use of SAR images from multiple views based on Bayesian theory-fused decision-making to obtain more reliable recognition results. Huan et al. first used support vector machine (SVM) to make independent decisions for each view and then proposed a decision fusion algorithm for different views based on the voting mechanism [38]. The other group believed that there were inherent connections between various views, which should be investigated. Zhang et al. first proposed a multiview SAR target recognition method based on joint sparse representation [39]. This method explored the internal associations between different views through joint sparse representation, thereby improving the accuracy of sparse representation of each view. Subsequently, many improved algorithms continued to appear under this framework, such as [40–42]. In theory, SAR images from different views of the same target must be related. However, due to the strong azimuth sensitivity of SAR images, the strength of this correlation is not stable enough. In other words, when the difference between the two views is small, the correlation between them is strong. Conversely, when the difference between the two views is large, the correlation between them is weak and not enough to reflect the internal correlation. Therefore, both independent inspection and joint representation are too arbitrary and cannot fully reflect the information contained in multiview SAR images.

Based on the above analysis, this paper fully examines the inner correlation in the multiview SAR images for target recognition. First, the correlation between two SAR images from different views is defined based on image correlation. On this basis, the correlation coefficient matrix between the multiview SAR images can be constructed to reflect the inherent correlation between different views. Then, the nonlinear correlation information entropy (NCIE) is used to calculate the correlations of a subset of the original multiview SAR images [44, 45]. The entropy represents the relevance of the selected viewing angle. Therefore, several multiview subsets with strong correlations can be obtained according to the resulted entropies. Finally, as a multitask processing algorithm, the joint sparse representation [39–41] is used for each set of SAR images to investigate the internal correlations of different views. It should be pointed out that there may only be one view in some collections, and the joint sparse representation will degenerate into the traditional sparse representation-based classification (SRC) at this time. For the decision value (reconstruction errors) output by each view set, the fusion errors are obtained in the form of linear weighting. And the target label of the multiview SAR image is determined according to the criterion of the smallest reconstruction error. In the experiment, the MSTAR dataset is used to comprehensively evaluate the proposed method. The result shows the effectiveness of the method in this paper.

## 2. Clustering of Multiview SAR Images

Due to the SAR imaging mechanism, the target image is closely related to the relative view angle between itself and the radar sensor. This leads to SAR images with strong azimuth sensitivity. In actual multiview SAR target recognition, the views involved in decision-making come from different azimuth angles; whole difference and interval are unknown. At this time, if the azimuth angles of some view angles are quite different, their inherent correlation is weak. And the joint sparse representation of them directly may introduce wrong constraints. Conversely, if the azimuth angles of some viewing angles are relatively close, there is a strong correlation between them. If they are arbitrarily analyzed independently, effective information will be lost. Therefore, it is necessary to preanalyze their internal relations and then adopt some appropriate classification algorithms.

### 2.1. Similarity Measure for SAR Images

Taking into account the independence and correlation between multiview SAR images, this study uses the idea of clustering to preprocess them. Specifically, the image correlation defined in equation (1) is used as the basic similarity measure for different views:

(1) $$\begin{array}{c} c=\max\left(\frac{\sum_{k}\sum_{l}\left[I_{1}\left(k,l\right)-m_{1}\right]\left[I_{2}\left(k-\Delta k,l-\Delta l\right)-m_{2}\right]}{{\left[\sum_{k}\sum_{l}{\left[I_{1}\left(k,l\right)-m_{1}\right]}^{2}{\left[I_{2}\left(k-\Delta k,l-\Delta l\right)-m_{2}\right]}^{2}\right]}^{\left(1/2\right)}}\right), \end{array}$$

where *I*_1_ and *I*_2_ represent two SAR images, *m*_1_ and *m*_2_ are their pixel averages, respectively, and Δ*k* and Δ*l* represent the sliding distance of the template image along the azimuth and distance directions. The correlation of SAR images is a prerequisite for multiview clustering in this study. In fact, there are many measures to evaluate the correlation of images. This study chooses a more classical image correlation coefficient, as shown in equation (1). The correlation coefficient examines the gray distribution difference between the two SAR images through pixel-level comparison. At the same time, the possible target registration errors are compensated by translation operations in two dimensions (range and azimuth). Therefore, the image correlation defined by equation (1) is more applicable in the evaluation of SAR image correlation in this study.

### 2.2. Clustering of Multiview SAR Images Based on NCIE

In order to effectively screen multiview SAR images reliably, this paper selects NCIE as the basic evaluation criterion for the internal correlation of different view sets [43, 44]. First, according to the similarity measure defined by equation (1), the correlation matrix between multiple views is constructed as follows:

(2) $$\begin{array}{c} R=E+\tilde{R}=\left[\begin{array}{cccc} 1 & r_{12} & \ldots & r_{1N}\\ r_{21} & 1 & \ldots & r_{2N}\\ \vdots & \vdots & \; & \vdots \\ r_{N1} & r_{N2} & \ldots & 1 \end{array}\right], \end{array}$$

where *E* is the identity matrix and $\tilde{R}$ represents the cross-correlation matrix between SAR images from *N* different views. According to the eigenvalues of *R*, the NCIE *H*_*R*_ is calculated as follows:

(3) $$\begin{array}{c} H_{R}=1+\sum_{t=1}^{T}\frac{\lambda_{t}}{T}\log_{T}\frac{\lambda_{t}}{T}. \end{array}$$

According to equation (3), when all views have completely different distributions, the correlation coefficient matrix is a unit one and all eigenvalues are 1. At this time, the NCIE is the minimum value 0. When the similarity between the viewing angles is greater than 0, the eigenvalues of the correlation coefficient matrix are no longer equal. When different viewing angles have exactly the same distribution, the NCIE is the maximum value of 1. Therefore, according to the values of the NCIE, the inner correlations between different combinations of views can be obtained.

In this study, the NCIE is used to select the optimal view sets. For the candidate SAR images with different views, they are combined according to predetermined rules to obtain *P* subset of different perspectives. Then, equation (3) is used to calculate the NCIE of each view set. Finally, according to the entropy sorting, the first *Q* view sets are selected for the subsequent multiview recognition algorithm.

## 3. Proposed Recognition Algorithm

### 3.1. Multitask Sparse Representation

Inspired by the theory of compressive sensing, sparse representation theory was developed and widely used in the field of pattern recognition [39–41]. The basic idea of SRC is to use multiclass training samples to linearly represent test samples with unknown target label while constraining that the representation coefficients have strong sparseness. Assuming that the test sample is *y*, the training sample are {*x*_1,1_, *x*_1,2_, ⋯*x*_1,*n*_1__ ⋯ *x*_*m*,1_, *x*_*m*,2_, ⋯, *x*_*m*,*n*_*m*__ ⋯ *x*_*M*,1_, *x*_*M*,2_, ⋯, *x*_*M*,*n*_*M*__}, where the subscripts 1∼*M* correspond to different training classes and *n*_*k*_ is the number of training samples of the *k*th class; the sparse representation can be described by the following equation:

(4) $$\begin{array}{c} y=\alpha_{1,1}x_{1,1}+\cdots \alpha_{m,n_{m}}x_{m,n_{m}}+\cdots \alpha_{M,n_{M}}x_{M,n_{M}}+\varepsilon , \end{array}$$

where {*α*_1,1_ ⋯ *α*_*m*,*n*_*m*__ ⋯ *α*_*M*,*n*_*M*__} is the sparse representation coefficient, that is, only a small number of the elements are nonzero.

The original SRC is mainly designed for a single signal or image. In fact, when there are multiple interrelated tasks for sparse representation at the same time, the correlation between them can be used to improve the accuracy of the representation. As a result, the joint sparse representation was proposed and applied. Let *K* related tasks be *y*=[*y*^(1)^,*y*^(2)^, ⋯ ,*y*^(*K*)^]; their joint sparse representation problem is as follows:

(5) $$\begin{array}{c} y^{\left(k\right)}=D^{\left(k\right)}\alpha^{\left(k\right)}+\varepsilon^{\left(k\right)},\;\left(k=1,2,\cdots ,K\right), \end{array}$$

where *y*^(*k*)^, *D*^(*k*)^, and *α*^(*k*)^ represent the test sample, training sample dictionary, and representation coefficient under the sparse representation task, respectively. In order to jointly solve the sparse representation coefficient vectors of multiple tasks, the optimization objective function shown in equation (6) can be used:

(6) $$\begin{array}{c} \underset{\beta }{\min}\left\{g\left(\beta \right)=\sum_{k=1}^{K}\left\|y^{\left(k\right)}-D^{\left(k\right)}\alpha^{\left(k\right)}\right\|\right\}, \end{array}$$

where *β*=[*α*^(1)^, *α*^(1)^  ⋯ *α*^(*K*)^] is a matrix containing the sparse coefficients.

Equation (6) ignores the correlation between different tasks in the optimization solution. In order to further improve the accuracy of the joint sparse representation, the sparse representation coefficient matrix can be appropriately constrained to make use of the associations seen in each task, as shown below:

(7) $$\begin{array}{c} \underset{\beta }{\min}g\left(\beta \right)+\lambda {\left\|\beta \right\|}_{2,1}, \end{array}$$

where ‖•‖_2,1_ denotes the *ℓ*_1_/*ℓ*_2_ norm. By adding this regular term, it is possible to constrain the sparse representations of different tasks to have similar sparse distribution characteristics (that is, the distribution law of nonzero coefficients), so as to explore the inherent relationship between them. To solve this problem, the algorithms in [39–41] can be used to solve the sparse representation coefficient matrix. Finally, the reconstruction error of each training class for multitask test samples can be calculated as follows:

(8) $$\begin{array}{c} f\left(i\right)=\sum_{k=1}^{K}\left\|y^{\left(k\right)}-D_{i}^{\left(k\right)}\alpha_{i}^{\left(k\right)}\right\|, \end{array}$$

where *D*_*i*_^(*k*)^ and *α*_*i*_^(*k*)^ represent the dictionary and sparse representation coefficients corresponding to the class under the *k*th task, respectively.

### 3.2. Recognition Procedure

In this study, a new classification strategy is designed for the target recognition problem of multiview SAR images. Through multiview clustering, the independence and correlation of multiview SAR images are effectively investigated. Then, the joint sparse representation is used to independently analyze each set of views with inherent correlation to obtain the reconstruction errors. The output reconstruction error of each view set is denoted as *f*_*t*_(*i*)(*t*=1,2, ⋯, *P*). Then, the linear weighting is used to fuse them as follows:

(9) $$\begin{array}{c} e\left(i\right)=\omega_{1}f_{1}\left(i\right)+\omega_{2}f_{2}\left(i\right)+\cdots +\omega_{P}f_{P}\left(i\right), \end{array}$$

where *ω*_*i*_(*i*=1,2, ⋯, *P*) is the weight coefficient. In this study, the weight is determined according to the number of SAR images in each view set as *ω*_*i*_=(*p*_*i*_/*P*), where *p*_*i*_ is the number of images in the *i*th view set. Finally, the target label is determined according to the weighted reconstruction error of each training class.

**Figure 1:**
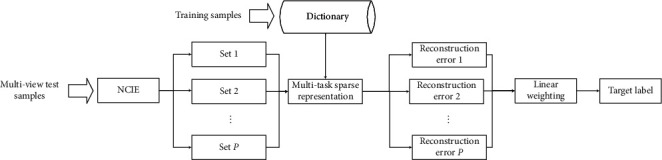
Procedure of target recognition based on clustering and joint representation of multiview SAR images.

Figure 1 shows the basic process of the method in this paper, including multiview clustering, multiview joint sparse representation, and linear weighted fusion of the results of each view set. The final recognition performance can be improved by examining the independence and correlation of multiview SAR images. In the specific implementation, in order to reduce the dimensionality of the SAR images, a random projection algorithm is used to transform it into a 520-dimensional feature vector according to [39]. It is worth noting that when some clustering results contain only one SAR image, the joint sparse representation degenerates to the traditional SRC, which does not affect the implementation of the proposed method.

## 4. Experiments

### 4.1. MSTAR Dataset

**Figure 2:**
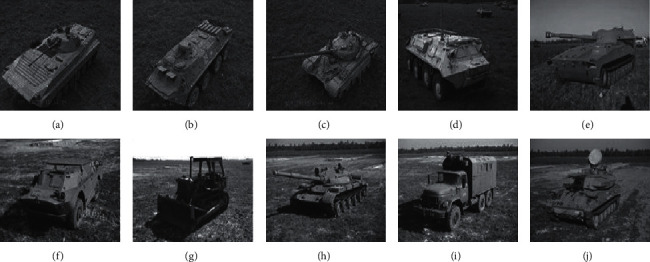
Images of the 10 targets for experiments. (a) BMP2 (b) BTR70 (c) T72 (d) BTR60 (e) 2S1, (f) BRDM2 (g) D7 (h) T62 (i) ZIL131 (j) ZSU23/4.

This study uses the SAR images of ten types of MSTAR targets (the optical images are shown in Figure 2) to test the proposed method. All the SAR images have the same resolution of 0.3 m × 0.3 m. A typical experimental setup is listed in Table 1. Among them, the training samples are collected from a 17° depression angle, and the test samples are collected from a 15° depression angle. In addition, by some simulation algorithms such as noise addition and occlusions, more experimental conditions can be generated to further evaluate the proposed method.

For comparison, this paper sets up several types of reference methods, including both the traditional single-view ones and the recently published multiview SAR target recognition ones. The single-view SAR target recognition method based on the SRC in [23] and the convolutional neural network (CNN)-based method in [28] are selected as the references. In the SRC method, the random projection is used to reduce the original SAR image to a 520-dimensional feature vector, which is consistent with the method in this paper. The CNN method is directly based on the original gray value of the SAR image for training and classification. The multiview methods in [38, 39] are selected for comparison, and they are recorded as “parallel multiview” and “joint multiview” methods, respectively. The parallel multiview method considers that each perspective is independent of each other, and makes decisions for each perspective and finally merges it. The joint multiview method is considered that multiview SAR images are inherently related, so they are unified and jointly expressed, and the target label is determined according to the sum of the final reconstruction errors.

### 4.2. Results and Analysis

#### 4.2.1. Preliminary Validation

**Figure 3:**
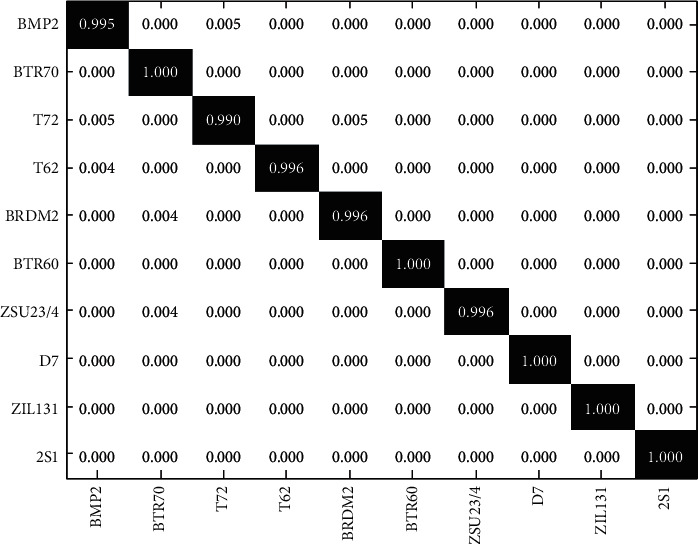
Results of 10-class recognition achieved by the proposed method.

First, the proposed method is tested under the conditions of a fixed number of views. When the number of views is set to 3 and the azimuth angle interval of different viewing angles is 2°, the recognition results of the proposed method on the 10 types of targets under the conditions of Table 1 can be expressed as the confusion matrix shown in Figure 3. It can be seen that the correct recognition rates of various targets (corresponding to the diagonal elements in the figure) is above 98.5%, and the average recognition rate is 99.42%.  Table 2 shows the average recognition rates of various reference methods under current conditions. In general, since the target information provided by multiview SAR images is more comprehensive, the recognition rates of the three types of multiview methods maintain a relatively high level and outperform the single-view ones. CNN can obtain strong classification ability when the training samples are sufficient, so it has also achieved high recognition performance. The comparison results show that the method in this paper is the most effective for 10 types of target recognition problems.

#### 4.2.2. Performance at Different Numbers of Views

**Figure 4:**
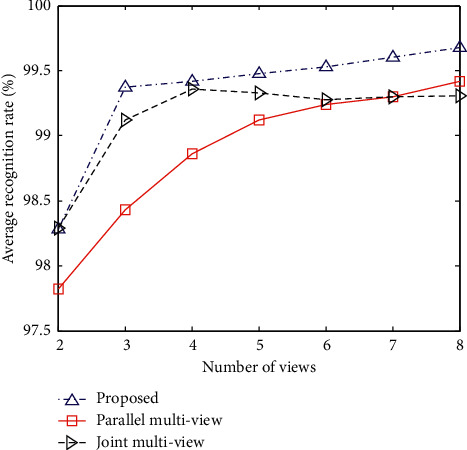
The recognition performance of the multiview methods at different view numbers.

The number of views is an important parameter in the multiview SAR target recognition method. For this reason, this experiment tests the recognition performance of the proposed method under different view angles. When the azimuth interval of different viewing angles is set to 2°, the average recognition rates of the three types of multiview methods varies with the viewing angle, as shown in Figure 4. The recognition performance of the method in this paper is increasing with the increase of the number of viewing angles. For the parallel multiview method, the recognition rate is also increasing, but the overall level remains relatively low. In the joint multiview method, when the number of views is less than 4, the recognition rate keeps increasing. However, when the number of viewing angles is large, the recognition rate decreases slightly. This is mainly because, at this time, a part of the viewing angles has relatively weak internal correlation due to the large azimuth angle difference, which leads to a decrease in the accuracy of the joint sparse representation.

#### 4.2.3. Noise Corruption

**Figure 5:**
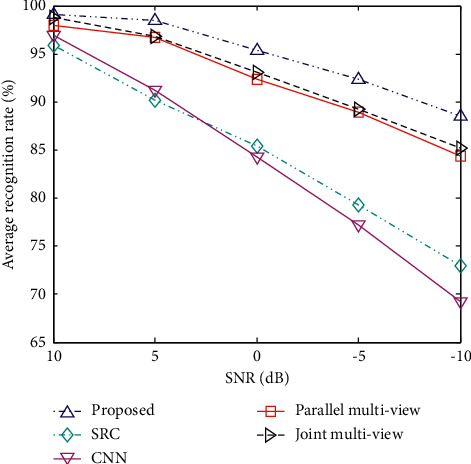
The recognition performance of different methods at different SNRs.

The recognition performance of the proposed method under noise interference conditions is investigated by adding different degrees of Gaussian white noise to the test samples in Table 1. The specific noise adding process can be found in [17].  Figure 5 shows the recognition performance of various methods under noise interference conditions. Among them, the number of viewing angles of the multiview method is set to 3, and the azimuth angle interval is 2°. The method in this paper maintains the highest average recognition rate under each signal-to-noise ratio (SNR), verifying its robustness to noise corruption. Compared with single-view methods, the performance of the three types of multiview methods is better, mainly due to the fact that multiview SAR images can provide more effective information for classification. The method in this paper further improves the robustness of the recognition method against noise interference by carefully examining the independence and correlation of multiview SAR images.

## 5. Conclusion

This paper proposes a multiview SAR target recognition method. For the multiple views involved in decision-making, their inner correlations are first analyzed based on image correlation and NCIE. Several view sets with strong internal correlations are obtained. Then, the joint sparse representation is used to investigate the internal association of the SAR images in each view set. Finally, a more robust decision variable is obtained through linear weighted fusion, so as to determine the target label more accurately. The experimental results based on the MSTAR dataset show that the method in this paper can achieve good recognition performance under a variety of experimental conditions, verifying its effectiveness.

## Figures and Tables

**Table 1:** A typical experimental setup based on the MSTAR dataset.

Class	Training (17°)	Test (15°)
BMP2	233	195
BTR70	233	196
T72	232	196
T62	299	273
BRDM2	298	274
BTR60	256	195
ZSU23/4	299	274
D7	299	274
ZIL131	299	274
2S1	299	274

**Table 2:** Average recognition rates achieved by different methods.

Method type	Average recognition rate (%)
Proposed	99.42
SRC	97.68
CNN	99.06
Parallel multiview	99.12
Joint multiview	99.30

## Data Availability

The dataset used to support the findings of the study are obtained from the corresponding author upon request.
